# Pathogen presence, prevalence, and diversity in *Ixodes scapularis* and mammal hosts at their expanding northern range limits

**DOI:** 10.3389/fpara.2023.1272790

**Published:** 2024-01-11

**Authors:** Kirsten E. Crandall, Jeremy T. Kerr, Virginie Millien

**Affiliations:** ^1^ Department of Biology, University of Ottawa, Ottawa, ON, Canada; ^2^ Department of Biology, McGill University, Montreal, QC, Canada; ^3^ Redpath Museum, McGill University, Montreal, QC, Canada

**Keywords:** pathogen, *Ixodes scapularis*, mammal hosts, *Peromyscus leucopus*, abundance, diversity

## Abstract

With climate and land use changes, tick-borne pathogens are expected to become more widely distributed in Canada. Pathogen spread and transmission in this region is modulated by changes in the abundance and distribution of tick and host populations. Here, we assessed the relationships between pathogens detected in *Ixodes scapularis* and mammal hosts at sites of different levels of disease risk using data from summer field surveys in Ontario and Quebec, Canada. Generalized linear mixed models and ordinal logistic regressions were used to determine the influence of the abundance of *I. scapularis* and the abundance and diversity of mammal hosts on pathogen presence, prevalence, and diversity. We detected three pathogen species in *I. scapularis* and small mammals using nested PCRs, namely *Borrelia burgdorferi* sensu stricto, *Babesia odocoilei*, and *Babesia microti*. Depending on the analyzed pathogen, local infection prevalence ranged from 0% to 25.4% in questing ticks and from 0% to 16.7% in small mammal hosts. We detected *B. odocoilei* in localities beyond its known range limits in southeastern Quebec suggesting ongoing range expansion of this pathogen. Neither the abundance of *I. scapularis* nor the abundance and diversity of mammal hosts altered local pathogen presence and prevalence, contrary to expectations. However, mammal species richness was a key predictor of the number of pathogen species. Our study demonstrates the need for future surveillance efforts that test questing and feeding *I. scapularis* of all life stages, as well as their hosts to better determine the spread, transmission, and co-occurrence of tick-borne pathogens in Canada.

## 1 Introduction

Tick-borne pathogens have increased in prevalence and geographic range in Canada due to changes in the abundance and distribution of tick and host populations (Ogden and Lindsay, 2016; Bouchard et al., 2019). Host populations are expanding their geographic ranges poleward in response to changes in climate and land use, thereby dispersing tick vectors to new poleward locations in Canada (Diuk-Wasser et al., 2021). As a result, reproducing tick populations may become established, which may subsequently increase tick-borne pathogen spread and transmission locally (Milnes et al., 2019). Consequently, increased tick abundances as well as increased prevalences and co-occurrences of tick-borne pathogens may lead to a greater number of cases of tick-borne diseases and co-infections in human populations (Cutler et al., 2021).

The predominant tick-borne pathogen detected via sentinel surveillance in Canada is *Borrelia burgdorferi* sensu stricto, one of the *Borrelia* genospecies that causes Lyme disease (Guillot et al., 2020; Wilson et al., 2022). This pathogen is transmitted by blacklegged ticks (*Ixodes scapularis*) in central and eastern Canada, as well as western blacklegged ticks (*I. pacificus*) in British Columbia (Guillot et al., 2020; Wilson et al., 2022). In these regions, the prevalence of *B. burgdorferi* in nymph and adult *Ixodes* ticks ranges from 0% to 56.0%, with the highest infection prevalences documented in Ontario, Quebec, New Brunswick, and Nova Scotia (Guillot et al., 2020; Dumas et al., 2022).

However, additional emerging tick-borne pathogens have been detected at a lower prevalence in *Ixodes* ticks through surveillance efforts in Canada (Dibernardo et al., 2014; Guillot et al., 2020; Wilson et al., 2022). *Anaplasma phagocytophilum*, the bacterium causing anaplasmosis, has been found in *I. scapularis* in Ontario, Quebec, New Brunswick, and Nova Scotia (Guillot et al., 2020). *Babesia microti*, a protozoan causing babesiosis, has also been identified in localities in British Columbia, Ontario, Quebec, New Brunswick, and Nova Scotia (Guillot et al., 2020; Wilson et al., 2022). Similarly, *Babesia odocoilei* has been found more recently in *I. scapularis* in Ontario and Quebec (Milnes et al., 2019; Scott and Pesapane, 2021; Crandall et al., 2022). *Borrelia miyamotoi*, a bacterium causing tick-borne relapsing fever, has been found at a low prevalence in *I. scapularis* in Ontario and Quebec (Guillot et al., 2020; Dumas et al., 2022).

The prevalence and transmission of tick-borne pathogens may be modulated by the abundance and composition of mammal communities (Levi et al., 2016; Luis et al., 2018). Small mammal hosts, such as white-footed mice (*Peromyscus leucopus*), chipmunks (*Tamias striatus*), and shrews (*Blarina brevicauda* and *Sorex cinereus*), can successfully feed a greater number of ticks and more readily transmit pathogens including *B. burgdorferi* (Mather et al., 1989; LoGiudice et al., 2003). In addition, mid-size or larger mammals, such as raccoons (*Procyon lotor*) and white-tailed deer (*Odocoileus virginianus*), feed large burdens of ticks resulting in increased tick abundances, yet these hosts may not be as efficient in transmitting pathogens (LoGiudice et al., 2003). In Ontario and Quebec, mammal species richness and the relative abundance of *P. leucopus* were both identified as significant contributors to increased *I. scapularis* abundance and *B. burgdorferi* prevalence, demonstrating the importance of the host community composition for pathogen spread and transmission (Simon et al., 2014; Werden et al., 2014; Dumas et al., 2022; Millien et al., 2023).

Tick and mammal host populations have been identified as key contributors to the spread and transmission of emerging tick-borne pathogens in Canada (Ogden et al., 2013; Bouchard et al., 2019). However, the degree that *I. scapularis* and mammal hosts impact tick-borne pathogen spread and transmission remains uncertain relative to their time since establishment (Millien et al., 2023). Here, we assessed the relationships between local pathogen presence, prevalence, and diversity with the abundance of *I. scapularis* as well as the abundance and diversity of mammal hosts at sites of distinct levels of disease risk in Ontario and Quebec, Canada. These results add knowledge of biotic factors that may help explain tick-borne pathogen spread and transmission at their frontier of range expansion in Canada.

## 2 Materials and methods

### 2.1 Field sampling

**Figure 1:**
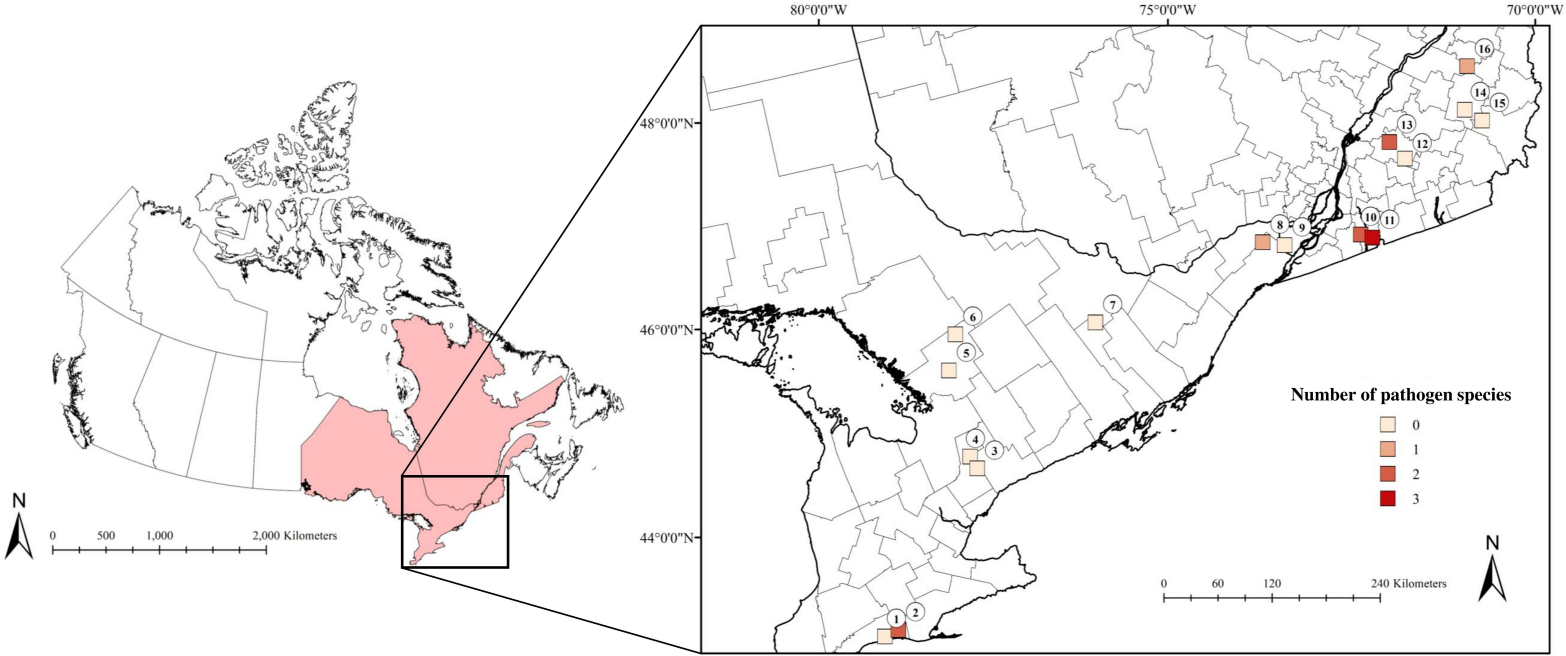
The number of pathogen species present at our sites in Ontario and Quebec, Canada that were detected in *Ixodes scapularis* and small mammals collected in July and August 2019. Lighter shades correspond to low numbers of pathogen species, while darker shades indicate a higher number of pathogen species. (1) 3 Ridges Farm, (2) New New Age Farm, (3) North Tract, (4) Brown Hill Tract, (5) Upjohn Nature Reserve, (6) Dyer Memorial Nature Reserve, (7) Rose Hill Nature Reserve, (8) Kirkview Farm, (9) Saint-Polycarpe, (10) Saint-Valentin, (11) Henryville, (12) Lefebvre, (13) Parc du Sanctuaire Saint-Majorique, (14) Serpentine-de-Coleraine Ecological Reserve, (15) Frontenac National Park, (16) Saint-Sylvestre.

Field surveys were conducted at 16 sites with contiguous forest in Ontario and Quebec, Canada in July and August 2019 (**Figure 1**). Sites were selected based on their different degrees of *B. burgdorferi* risk related to the abundances and life stages of *I. scapularis* present locally as defined by the Institut national de santé publique du Québec (2018) and Public Health Ontario (2018), which ranged from possible to significant risk (**Supplementary Table 1**). At each site, three grids of 40 m by 70 m were set up for sampling ticks and mammal hosts, which were maximally separated by 100 meters due to geographic barriers (e.g., streams or park trails).

Within each grid, four 70-meter long transects were used to sample ticks one time by dragging a 1 m^2^ white flannel over low-lying vegetation. Flannels were checked every 10 meters, and questing ticks were removed. All ticks were kept in microvials with 95% ethanol, and larvae were pooled while nymphs and adults were kept individually. Tick specimens were identified to the species using dichotomous keys (Lindquist et al., 2016).

At each site, 84 Sherman live traps (H.B. Sherman Traps, Inc., Florida, United States) were placed along four parallel transects within each grid for three consecutive nights, representing a total of 4032 trap nights in our study. We targeted mouse (*P. leucopus* and *P. maniculatus*), shrew (*B. brevicauda* and *S. cinereus*), vole (*Microtus pennsylvanicus* and *Myodes gapperi*), and jumping mouse (*Napaeozapus insignis* and *Zapus hudsonius*) species. In the afternoon, a bait mixture of peanut butter and oatmeal, an apple piece, and a cotton ball were placed in each trap. Traps were checked the following morning. Juveniles and non-targeted rodent species were immediately released at the site of capture. Individuals of targeted species were euthanized via isoflurane inhalation followed by cervical dislocation. One red squirrel (*Tamiasciurus hudsonicus*) and two hairy-tailed moles (*Parascalops breweri*) were also euthanized due to severe injuries. Small mammals were searched for feeding ticks, and mammalian liver tissues were dissected and placed into microvials with 95% ethanol. Liver tissues were selected for pathogen testing, as they have been used for *B. burgdorferi* detection in wild rodents (Zinck and Lloyd, 2022). As in Tessier et al. (2004), a nested PCR using species-specific COIII primers was used to identify *Peromyscus* species (**Supplementary Methods**). All samples were accessioned in the collections of the Redpath Museum, McGill University (**Supplementary Table 2**). Ethical approval and permits were issued by McGill University (AUP No. 2019-8086), the Ministère des Forêts, de la Faune et des Parcs (SEG permit No. 2019-06-04-008-00-S-F), and the Ministry of Natural Resources and Forestry (WSCA No. 1093495).

Concurrently, nine trail cameras (Force-10, SpyPoint Inc., Quebec, Canada) were placed 1 meter above the ground facing inside our grids and set to take three consecutive photos without delay for each detection. Host species were identified from photographs taken by camera traps. Birds, domestic pets, humans, and unidentified individuals were not included in our dataset.

For each site, total *I. scapularis* abundance was estimated by the sum of questing and feeding ticks collected from tick dragging and small mammal trapping, respectively. We used the total number of collected mammal individuals as a proxy for the abundance of small mammals locally. The relative abundance of *P. leucopus* was quantified as the number of collected *P. leucopus* individuals divided by the local abundance of collected small mammals. The number of mammal host species was estimated as the number of distinct species collected via small mammal trapping and detected in camera photographs.

### 2.2 Pathogen testing

DNA extractions and nested PCRs conducted by Geneticks, Inc. targeted five pathogens in our tick and small mammal specimens (**Supplementary Methods**; **Supplementary Table 3**). Adults and nymphs were tested individually, while larvae were pooled by grid if questing (2-10 larvae per pool) and by host if feeding (1-10 larvae per pool). All *I. scapularis* and small mammal specimens were tested for *Anaplasma phagocytophilum*, *Babesia* species, and *Borrelia* species. If a band was visible (i.e., positive PCR), we then tested twice more for false positives. The *p44* gene was targeted to test for *A. phagocytophilum* (Holden et al., 2003). *Babesia odocoilei* and *B. microti* were targeted with the 18S rRNA region using the *mic494* and *odo563* inner primers, respectively. An additional primer set targeting the 18S rRNA of each *Babesia* species was used for confirmation (Persing et al., 1992). We also tested for *B. burgdorferi* sensu stricto and *B. miyamotoi* using the 5S-23S intergenic space region and the 18S rRNA region, respectively (Dibernardo et al., 2014; Zinck et al., 2021). An additional test using the *flaB* gene confirmed the presence of *B. burgdorferi* sensu lato (Wodecka, 2011). Bio Basic DNA Sequencing (Ontario, Canada) completed Sanger DNA sequencing of positive samples, with sequences assessed for quality control, ambiguous base calls, and end-reading errors using 4Peaks software. Pathogen species were confirmed with GenBank using a MEGABLAST search in the nucleotide BLAST database.

For each site, we calculated pathogen presence, prevalence, and diversity in *I. scapularis* and small mammal hosts. Pathogen presence indicated whether pathogens were present (1) or absent (0) locally in *I. scapularis* or in small mammal hosts. Pathogen prevalence was calculated as a proportion for questing *I. scapularis* by dividing the number of infected individuals and larval pools of *I. scapularis* by the total number of *I. scapularis* (individuals and larval pools). Feeding *I. scapularis* were excluded from this calculation, as they better represent the pathogens circulating in hosts and may artificially increase local infection prevalence. Pathogen diversity was defined as the total number of pathogen species found in *I. scapularis* and small mammal hosts.

### 2.3 Statistical analyses

All statistical analyses were performed in R v4.2.2. (R Core Team, 2022). We assessed the effect of *I. scapularis* and mammal hosts on pathogen presence, prevalence, and diversity across our sites in Central Canada. Using the *rcorr* function in the *Hmisc* package (Harrell Jr, 2021), we first calculated the correlation coefficients between small mammal abundance, the relative abundance of *P. leucopus*, mammal species richness, questing *I. scapularis* abundance, and total *I. scapularis* abundance. Small mammal abundance was highly correlated with mammal diversity (r = 0.54, *p* < 0.05), and was not included in further analyses. Using the *scale* function, biotic factors were centered by subtracting the variable average from each value and standardized. Spatial autocorrelation among biotic factors was assessed with Moran’s I with an inverse distance weights matrix using the *moran.test* function in the *spdep* package (Bivand and Wong, 2018).

We first evaluated the effect of *I. scapularis* abundance and mammal hosts on pathogen presence with two binomial generalized linear mixed models with a cloglog link function using the *glmer* function in the *lme4* package (Bates et al., 2015). Our binary response variable was pathogen presence (1) or absence (0) in *I. scapularis* and small mammal hosts. Two separate models were run to determine the independent impacts that *I. scapularis* and mammal hosts have on pathogen presence. The first model used the total *I. scapularis* abundance as an independent variable. The independent variables of a second model included the relative abundance of *P. leucopus* and mammal species richness. We subsequently analyzed the impact of mammals hosts on pathogen prevalence in questing *I. scapularis* with a binomial generalized linear mixed model and a cloglog link function using the *glmer* function. A binomial model was used, as pathogen prevalence was calculated as a proportion. The independent variables in this third model were the relative abundance of *P. leucopus* and mammal species richness. Site was included as a random factor in all three models to account for spatial autocorrelation. Model selection was based on AIC values, with a smaller AIC indicating a better model fit, and the variance and standard deviation of Site.

Finally, we ran an ordinal logistic regression with the *polr* function in the MASS package (Venables and Ripley, 2002) to assess if pathogen diversity across our sites was affected by mammal hosts. Our independent variables included the relative abundance of *P. leucopus* and the number of mammal host species. We used the *stepAIC* function in the *cAIC4* package (Säfken et al., 2021) to determine if additional models should be assessed.

## 3 Results

### 3.1 Field sampled ticks and mammal species

The abundance of questing and feeding *I. scapularis* ranged from 0 to 164 individuals across our sites (**Tables 1**, **2**). We collected a total of 382 questing *I. scapularis* including 255 larvae (29 pools), 126 nymphs, and one adult male, as well as 65 feeding *I. scapularis* including 57 larvae (17 pools) and 8 nymphs.

**Table 1:** The number of infected and total *Ixodes scapularis* at our study sites in Ontario and Quebec, Canada.

Site ID	Larval pools	Nymphs	Adults	Pathogen species identified in ticks
1	0	0	0	None
2	Q: 0/12 F: 0/7	Q: 2/15 F: 0/2	0	*B. burgdorferi* (1 nymph), *B. odocoilei* (1 nymph)
3	0	F: 0/2	0	None
4	0	0	0	None
5	0	0	0	None
6	0	0	0	None
7	0	0	0	None
8	0	Q: 0/4 F: 1/1	0	*B. burgdorferi* (1 nymph)
9	F: 0/1	Q: 0/1	0	None
10	Q: 0/4 F: 0/4	Q: 19/67 F: 0/1	0	*B. burgdorferi* (18 nymphs), *B. odocoilei* (1 nymph)
11	Q: 0/10 F: 1/3	Q: 4/26 F: 0/2	Q: 0/1	*B. burgdorferi* (3 nymphs), *B. odocoilei* (1 nymph, 1 larva)
12	0	0	0	None
13	Q: 0/2 F: 1/2	Q: 2/12	0	*B. burgdorferi* (1 larva), *B. odocoilei* (2 nymphs)
14	Q: 0/1	Q: 0/1	0	None
15	0	0	0	None
16	0	0	0	None

Tick abundances consisted of larval pools between 1 to 10 larvae, individual nymphs, or individual adults. Questing ticks are denoted by a “Q” and ticks feeding on small mammals are indicated by an “F”. Pathogens detected in ticks included *Babesia odocoilei* and *Borrelia burgdorferi*. See **Supplementary Table 6** for details regarding infection prevalence by tick activity and pathogen species. (1) 3 Ridges Farm, (2) New New Age Farm, (3) North Tract, (4) Brown Hill Tract, (5) Upjohn Nature Reserve, (6) Dyer Memorial Nature Reserve, (7) Rose Hill Nature Reserve, (8) Kirkview Farm, (9) Saint-Polycarpe, (10) Saint-Valentin, (11) Henryville, (12) Lefebvre, (13) Parc du Sanctuaire Saint-Majorique, (14) Serpentine-de-Coleraine Ecological Reserve, (15) Frontenac National Park, (16) Saint-Sylvestre.

**Table 2:** Summary of pathogen presence, prevalence, diversity in addition to the abundance and diversity of *Ixodes scapularis* and mammal hosts found at each site in Ontario and Quebec, Canada (listed as increasing latitudes and decreasing longitudes).

Site ID	Latitude (°N)	Longitude (°W)	Abundance *I. scapularis*	Pathogen presence	Pathogen diversity	Pathogen prevalence	Abundance small mammals	Relative abundance *P. leucopus*	No. host species (trapping and camera)
1	42.70	-81.03	0	0	0	0.000	2	0.500	7
2	42.73	-80.84	164	1	2	0.074	18	0.333	7
3	44.08	-79.31	2	0	0	0.000	6	0.167	5
4	44.21	-79.36	0	0	0	0.000	5	1.000	3
5	45.08	-79.36	0	0	0	0.000	2	1.000	3
6	45.40	-79.15	0	0	0	0.000	2	0.000	3
7	45.16	-77.22	0	0	0	0.000	7	0.000	5
8	45.42	-74.67	5	1	1	0.000	5	1.000	6
9	45.33	-74.39	2	0	0	0.000	13	0.615	5
10	45.18	-73.35	117	1	2	0.268	9	0.333	5
11	45.12	-73.21	125	1	3	0.108	6	0.333	7
12	45.74	-72.41	0	0	0	0.000	5	0.000	5
13	45.94	-72.53	29	1	2	0.143	10	0.000	8
14	45.98	-71.37	3	0	0	0.000	4	0.000	2
15	45.82	-71.20	0	0	0	0.000	1	0.000	4
16	46.37	-71.12	0	1	1	0.000	10	0.000	6

Total *I. scapularis* abundance represents the abundance of questing and feeding ticks at each site found via tick dragging and small mammal trapping, respectively. Pathogen presence indicated whether pathogens were present (1) or absent (0) in *I. scapularis* or in small mammal hosts at a locality. Pathogen diversity is the number of tick-borne pathogen species found at each site in *I. scapularis* and small mammals. Pathogen prevalence was calculated as a proportion with questing *I. scapularis* by dividing the number of infected individual *I. scapularis* and larval pools by the total *I. scapularis* (individuals and larval pools). The relative abundance of *Peromyscus leucopus* was estimated by dividing the number of collected *P. leucopus* individuals by the local abundance of small mammals that were collected. The number of mammal host species was quantified as the sum of the different host species found via small mammal trapping and in trail camera photographs. (1) 3 Ridges Farm, (2) New New Age Farm, (3) North Tract, (4) Brown Hill Tract, (5) Upjohn Nature Reserve, (6) Dyer Memorial Nature Reserve, (7) Rose Hill Nature Reserve, (8) Kirkview Farm, (9) Saint-Polycarpe, (10) Saint-Valentin, (11) Henryville, (12) Lefebvre, (13) Parc du Sanctuaire Saint-Majorique, (14) Serpentine-de-Coleraine Ecological Reserve, (15) Frontenac National Park, (16) Saint-Sylvestre.

We collected a total of 105 small mammal individuals (**Table 2**; **Supplementary Table 4**, **5**). The most abundant species was *P. leucopus* (31.4% of collected individuals; *n* = 33), which was present at 9 of 16 sites. Other collected small mammals, in decreasing order of abundance, were *N. insignis* (22.8%; *n* = 24), *B. brevicauda* (16.2%; *n* = 17), *M. gapperi* (15.2%; *n* = 16), *P. breweri* (1.9%; *n* = 2), *S. cinereus* (1.0%; *n* = 1), and *M. pennsylvanicus* (1.0%; *n* = 1). At each site, small mammal abundance ranged from 1 to 18 individuals, while the relative abundance of *P. leucopus* ranged from 0 to 1 (**Table 2**). We identified 5 mammal species in photographs taken by cameras, which included squirrels (*Sciurus carolinensis*), chipmunks (*T. striatus*), white-tailed deer (*O. virginianus*), raccoons (*P. lotor*), and coyotes (*Canis latrans*). Between 2 and 8 mammal species were detected at each site via small mammal trapping and in camera photographs.

### 3.2 Pathogen diversity

For questing *I. scapularis*, only nymphs tested positive for our pathogens of interest, while the 255 questing larvae (29 pools) and one adult male that we collected all tested negative. Of 126 *I. scapularis* nymphs, five tested positive for *B. odocoilei*, with one nymph at Site 2, Site 10, and Site 11 and two nymphs at Site 13 (**Tables 1**, **2**). Similarly, *I. scapularis* nymphs infected with *B. burgdorferi* were found at Site 2 (one nymph), Site 10 (18 nymphs), and Site 11 (three nymphs). Local infection prevalence in questing *I. scapularis* ranged from 0% to 14.3% for *B. odocoilei* and from 0% to 25.4% for *B. burgdorferi* (**Supplementary Table 6**). No questing ticks tested positive for *A. phagocytophilum*, *B. microti*, or *B. miyamotoi*.

For feeding *I. scapularis*, 2 of 17 larval pools and 1 of 8 nymphs were infected (**Tables 1**, **2**; **Supplementary Table 6**). One larva at Site 11 was infected with *B. odocoilei*, but it was found feeding on a *P. leucopus* infected with *B. microti*. A larva from Site 13 feeding on an uninfected *P. maniculatus* and a nymph from Site 8 feeding on an uninfected *P. leucopus* were both infected with *B. burgdorferi*. No feeding ticks tested positive for *A. phagocytophilum*, *B. microti*, or *B. miyamotoi*.

Only two of the 105 small mammals that we collected were infected (**Supplementary Tables 4**, **5**). At Site 11, one *P. leucopus* was infected with *B. microti* and at Site 16, one *B. brevicauda* also tested positive for *B. odocoilei*. Local infection prevalence of small mammal hosts ranged from 0% to 10% for *B. odocoilei* and from 0% to 16.7% for *B. microti* (**Supplementary Table 7**). No small mammals tested positive for *A. phagocytophilum*, *B. burgdorferi*, or *B. miyamotoi*.

We detected pathogens in *I. scapularis* and small mammals hosts at 6 of our 16 sites, where up to 3 different pathogen species were present locally (**Figure 1**; **Tables 1**, **2**). Local pathogen prevalence in questing *I. scapularis* adults, nymphs, and pools of larvae ranged from 0% to 26.8% (**Table 2**). Pathogen diversity was found to be highest in areas with long-established populations of *I. scapularis* in southern Ontario and Quebec (Sites 2, 10, and 11). These sites had over 100 *I. scapularis* from at least two different life stages, where 2 or 3 pathogen species were detected locally (**Figure 1**; **Tables 1**, **2**).

### 3.3 Effect of biotic factors on pathogen presence, prevalence, and diversity

Small mammal abundance was significantly correlated to mammal species richness (r = 0.54, *p* < 0.05), questing *I. scapularis* abundance (r = 0.54, *p* < 0.05), and total *I. scapularis* abundance (r = 0.55, *p* < 0.05). Therefore, we excluded small mammal abundance from further analyses. We detected spatial autocorrelation in questing *I. scapularis* abundance (Moran’s I = 0.206, *p* < 0.05), but none was detected for the relative abundance of *P. leucopus* (Moran’s I = 0.072, *p* = 0.207) and mammal species richness (Moran’s I = 0.138, *p* = 0.115).

**Figure 2:**
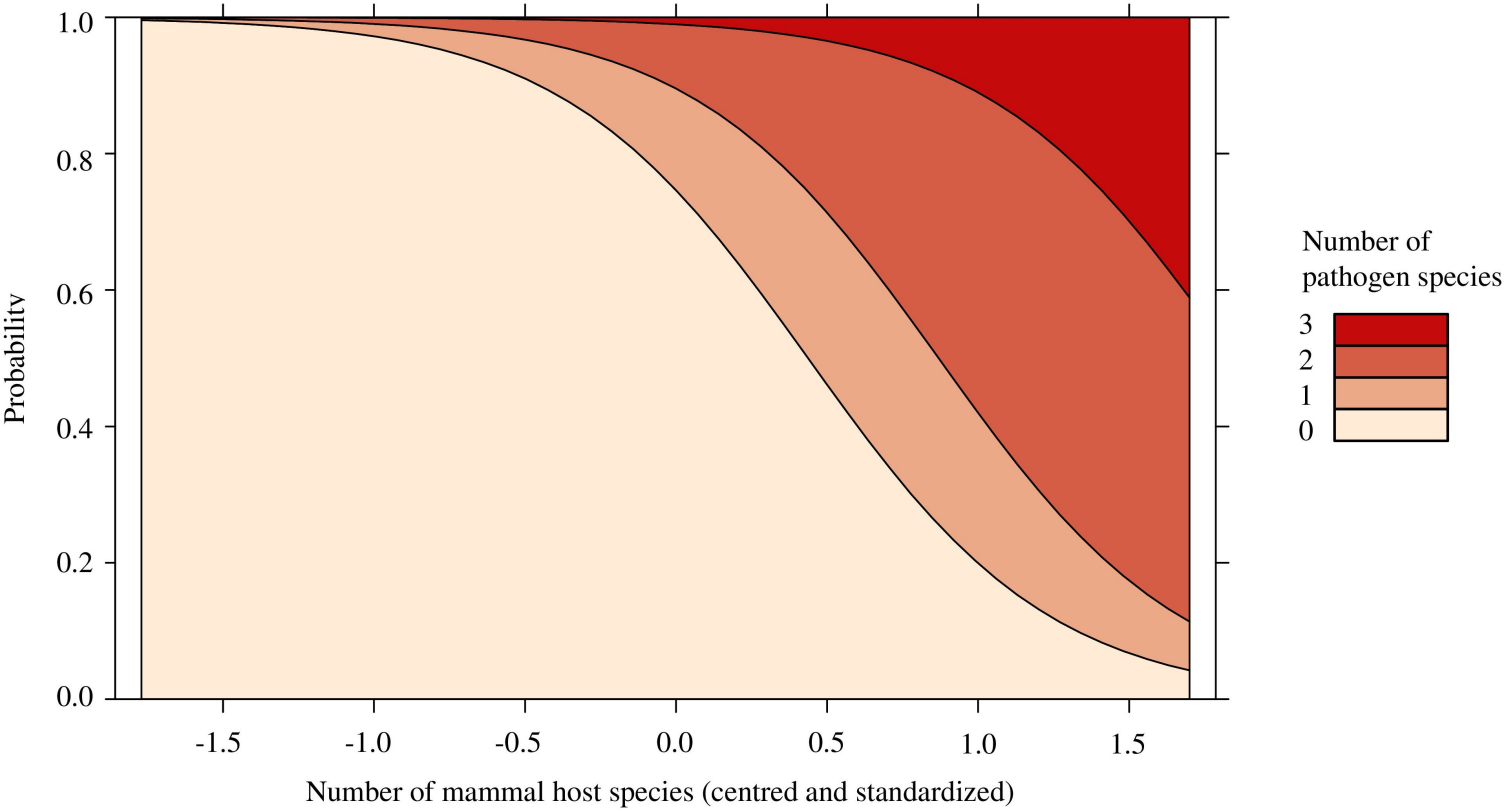
Stacked effect plot of the ordinal logistic regression predicting the number of pathogen species found in *Ixodes scapularis* and small mammals in Ontario and Quebec, Canada. The y-axis represents the probability that a certain number of pathogen species are present locally and the x-axis represents the centered and standardized values of mammal species richness, which ranged from 2 to 8 species. Local pathogen diversity is significantly predicted by the number of mammal host species found via small mammal trapping and trail cameras. As the number of mammal host species increased, the probability of presence and diversity of pathogens increased.

There was no effect of pathogen presence with total *I. scapularis* abundance (*p* = 0.144; **Supplementary Table 8**), the relative abundance of *P. leucopus* (*p* = 0.874; **Supplementary Table 9**), or mammal species richness (*p* = 0.192; **Supplementary Table 9**). Similarly, pathogen prevalence in questing *I. scapularis* was not affected by the relative abundance of *P. leucopus* (*p* = 0.824) or mammal species richness (*p* = 0.767; **Supplementary Table 10**). Feeding ticks were not included in this model, as to not artificially increase the local pathogen prevalence. Finally, the relative abundance of *P. leucopus* (*p* = 0.822) did not significantly predict pathogen diversity and was subsequently removed from our model (**Supplementary Table 11**). Only mammal species richness (OR = 11.826, *p* < 0.05) was found to significantly predict pathogen diversity, with higher odd ratios of pathogen detection with greater mammal species richness (**Figure 2**; **Supplementary Table 11**).

## 4 Discussion

We provide evidence that mammal host populations contribute to the local diversity of emerging tick-borne pathogens in Central Canada. Pathogen diversity was highest in areas with long-established populations of *I. scapularis* in southern Ontario and Quebec. Greater mammal species richness within study locations was associated with a greater diversity of pathogens. This relationship was detected using molecular techniques across this broad region, where pathogen presence and prevalence have been increasing. However, we did not find an effect of the abundance of *I. scapularis* nor the abundance and diversity of mammal hosts on local pathogen presence and prevalence. These results demonstrate the complex mechanism driving the poleward expansion and transmission of these tick-borne pathogens.

Local infection prevalence in questing ticks varied depending on pathogen species. In line with surveillance data, we found that the local infection prevalence of *B. odocoilei* was up to 14.3% in questing ticks (Milnes et al., 2019; Scott et al., 2020; Scott and Pesapane, 2021; Scott et al., 2021). Of note, we detected *B. odocoilei* in questing ticks in Saint-Majorique-de-Grantham and a shrew in Saint-Sylvestre, which are outside of its known range limit in Sainte-Anne-de-Bellevue, Quebec (**Supplementary Figure 1**; Scott and Pesapane, 2021). This ongoing range expansion may be facilitated by the dispersal of infected ticks by bird hosts that are known to be reservoirs for *B. odocoilei* (Scott et al., 2022). *B. burgdorferi* had the broadest geographic range amongst our tick samples, with infection prevalence highest in areas with long-established *I. scapularis* populations where the pathogen has been circulating for decades (Ogden et al., 2014). These results also parallel the infection rates reported at sentinel sites in Ontario and Quebec (Guillot et al., 2020).

### 4.1 The impact of biotic factors on pathogens

Abundances of key hosts, such as white-tailed deer and white-footed mice, have been associated with a greater *I. scapularis* abundance and greater *B. burgdorferi* prevalence in Ontario and Quebec (Bouchard et al., 2011; Bouchard et al., 2013; Simon et al., 2014; Werden et al., 2014). However, here, pathogen presence and prevalence were not affected by the abundance of *I. scapularis* nor the abundance and diversity of mammal hosts across our sites. Pathogen spread and transmission may have been affected by bird hosts, an unexplored factor in our study, due to their ability to feed immature ticks and harbor several tick-borne pathogens (Scott et al., 2022). Migratory birds may facilitate range expansions of tick-borne pathogens by introducing infected adventitious ticks to new locations in Canada (Scott et al., 2020; Scott and Pesapane, 2021). In addition, ground foraging birds were found to be significant contributors to the spread and transmission of tick-borne pathogens at the most northern parts of the distribution range of *I. scapularis* (Leo et al., 2017; Dumas et al., 2022).

We observed that mammal species richness significantly predicted pathogen diversity, with up to 3 pathogen species being detected locally (**Figures 1**, **2**). Sites with greater pathogen diversity were associated with more diverse mammal communities. Locally, small mammals play an important role in feeding immature ticks and pathogen maintenance (Mather et al., 1989; LoGiudice et al., 2003). As these small mammals search for food resources, they may disperse ticks and their pathogens over short distances into nearby forest patches (Marrotte et al., 2017; Borgmann-Winter et al., 2021). Larger mammal hosts, such as white-tailed deer, can feed large burdens of ticks and act as key reproductive hosts for adult *I. scapularis* (LoGiudice et al., 2003; Werden et al., 2014). These mammal hosts may act as important facilitators for the long-range dispersal and establishment of tick populations and tick-borne pathogens, as they expand their ranges poleward in response to climate and land use changes (Dawe and Boutin, 2016; Diuk-Wasser et al., 2021). However, the spread and transmission of tick-borne pathogens may be limited in areas where *I. scapularis* or reservoir hosts, such as *P. leucopus*, have not yet established (Roy-Dufresne et al., 2013; Simon et al., 2014; Ripoche et al., 2022; Millien et al., 2023). In these areas, other medically significant tick vectors (e.g., *Ixodes cookei* with Powassan virus; Gasmi et al., 2018) and reservoir hosts (e.g. chipmunks or shrews) may contribute more strongly to the spread and transmission of tick-borne pathogens (Levi et al., 2016; Gasmi et al., 2018; Dumas et al., 2022).

The co-occurrence of multiple tick-borne pathogens may increase the risk of co-infection in tick and host populations locally (Cutler et al., 2021). Co-infections can occur in adult ticks after feeding on different infected reservoir hosts or when co-feeding with infected ticks on the same host (Voordouw, 2015; Cutler et al., 2021). Although we did not detect any co-infections in our tick and small mammal specimens, co-infections have been detected at varying levels of infection prevalence in *I. scapularis* in Canada (Dibernardo et al., 2014; Dumas et al., 2022; Wilson et al., 2022). The majority of these co-infections occurred in areas near our sites in southern Ontario and Quebec, where long-established *I. scapularis* populations are located (Dumas et al., 2022; Wilson et al., 2022). If more tick-borne pathogens are co-occurring locally, it may lead to an increased risk of co-infections of tick-borne diseases in human populations. As a result, humans co-infected with multiple tick-borne pathogens may display complex clinical manifestations that present diagnostic challenges (Cutler et al., 2021). Although we detected no pathogen species at some sites, there does not appear to be any environmental or host suitability limitations in these areas that will prevent those tick-borne pathogens from spreading there in the future.

### 4.2 Future surveillance of tick-borne pathogens

Our study demonstrates that comprehensive surveillance efforts targeting questing and feeding *I. scapularis* of all life stages and small mammal hosts is required to detect the geographic extent and co-occurrence of tick-borne pathogens in Canada. Concurrent testing of multiple tick-borne pathogens is necessary to better detect the risk of co-infections, especially as the co-occurrence of pathogens become more prevalent in areas with increased tick abundances and more diverse host communities. These results show expanding ranges of certain tick-borne pathogens transmitted by *I. scapularis*, especially in areas where *B. burgdorferi* has not yet established. It would also be relevant to test questing larval *I. scapularis* for tick-borne pathogens with known transovarial transmission, such as *B. odocoilei* (Zembsch et al., 2021). Emerging tick-borne pathogens are advancing poleward in Canada with the expanding ranges of tick and host populations, where risks for pathogen transmission will rise. Proactive surveillance efforts outside the known distributions of pathogens of concern for wildlife and human health will improve our ability to better anticipate the risk for tick-borne diseases in these regions.

## Data availability statement

The original contributions presented in the study are included in the article/**Supplementary Material**. Further inquiries can be directed to the corresponding author.

## Ethics statement

The animal study was approved by McGill University (AUP No. 2019-8086), the Ministère des Forêts, de la Faune et des Parcs (SEG permit No. 2019-06-04-008-00-S-F), and the Ministry of Natural Resources and Forestry (WSCA No. 1093495). The study was conducted in accordance with the local legislation and institutional requirements.

## Author contributions

KEC: Conceptualization, Data curation, Formal analysis, Investigation, Methodology, Visualization, Writing – original draft, Writing – review & editing. JTK: Conceptualization, Funding acquisition, Supervision, Writing – review & editing. VM: Conceptualization, Funding acquisition, Supervision, Writing – review & editing.
