# Supplementary material for: Pathogen presence, prevalence, and diversity in Ixodes scapularis and mammal hosts at their expanding northern range limits
Source: Front Parasitol. 2024 Jan 11;2:1272790. doi: 10.3389/fpara.2023.1272790 (PMC11732163; doi:10.3389/fpara.2023.1272790)
Supplement: Supplementary file 1 [file DataSheet_1.docx]

**1. Supplementary Methods**

Geneticks Inc. conducted all DNA extractions and polymerase chain reactions (PCR). Adults and nymphs were tested individually, while larvae were pooled. Larvae that were questing were pooled by grid (2-10 larvae per pool), while those feeding were pooled by their host (1-10 larvae per pool). Ticks were homogenized using a microtube pestle in AquaGenomic solution (Wills et al. 2018). Samples were incubated in a 60°C heat block for 45 minutes, vortexed briefly, and centrifuged at 13,300 rpm for four minutes. The supernatant was removed and placed into a microvial with 50 µL isopropanol, which was inverted and centrifuged as before. After decanting the supernatant, the DNA pellet was rinsed with 50 µL of 70% ethanol. The sample was left to air dry for 15 minutes at room temperature and then resuspended with 50 µL of 1mM Tris pH 8.0. Samples were incubated in a 60°C heat block for one hour.

The Thermo Scientific GeneJET Genomic Purification Kit (Protocol A, 2016) (Thermo Fisher Scientific, Massachusetts, United States) was used to extract DNA from mammalian liver tissues. A few modifications were made to the Mammalian Tissue and Rodent Tail Genomic DNA Purification protocol. These modifications included the use of 10 mg of liver, an additional centrifugation at step nine to remove the residual solution, and no additional elution buffer was used after sitting for five minutes prior to centrifugation.

- 1. *Identification of Peromyscus species*

As in Tessier et al. (2004), a nested PCR using species-specific COIII primers was run to identify *Peromyscus* species. An initial denaturation time of 5 minutes was used as a modification.

- 1. *Pathogen screening in tick and mammal specimens*

Five pathogens were targeted in *Ixodes scapularis* and small mammal specimens using nested PCRs (see Table S2 for a description of the primers and conditions for each pathogen). All tick and small mammal specimens were tested for *Anaplasma phagocytophilum*, *Babesia microti*, *Babesia odocoilei*, *Borrelia burgdorferi*, and *Borrelia miyamotoi*. If a band was visible (i.e., positive PCR), we then tested twice more to identify false positives. The *p44* gene was used to test for the presence of *A. phagocytophilum* (Holden et al. 2003). *Babesia odocoilei* and *B. microti* were targeted with the 18S rRNA region using the *mic494* and *odo563* inner primers, respectively. An additional primer set targeting the 18S rRNA of each *Babesia* species was used for confirmation (Persing et al. 1992). We also tested for *B. burgdorferi* sensu stricto and *B. miyamotoi* using the 5S-23S intergenic space region and the 18S rRNA region, respectively (Dibernardo et al. 2014, Zinck et al. 2021). An additional test using the *flaB* gene confirmed the presence of *B. burgdorferi* sensu lato (Wodecka 2011).

- 1. *DNA sequencing and quality control*

Amplified products were purified for sequencing following Sun et al. (2012). A cotton cushion was placed in a 500 µL centrifuge tube after a hole was made in its bottom. An excised fragment of interest was removed from the agarose gel and laid on the cushion. The tube was capped and placed into an uncapped 1.7 mL Eppendorf tube, where samples were spun at 5000 rpm for 7 minutes. Purified DNA was then reamplified with the corresponding inner primers. Sanger DNA sequencing with forward inner primers was completed at Bio Basic DNA Sequencing (Markham, Ontario, Canada).

Using 4Peaks software (https://nucleobytes.com/4peaks/), sequences were assessed for quality control, ambiguous base calls, and end-reading errors. Our dataset consisted only of sequences with an average quality score of 20 or higher. We determined the pathogen species of our sequence with GenBank using a MEGABLAST search in the nucleotide BLAST database (https://blast.ncbi.nlm.nih.gov/Blast.cgi#_blank).

**References**

Dibernardo, A., T. Cote, N. Ogden, and L. Lindsay. 2014. The prevalence of *Borrelia miyamotoi* infection, and co-infections with other *Borrelia* spp. in *Ixodes scapularis* ticks collected in Canada. Parasites & Vectors 7.

Holden, K., J. T. Boothby, S. Anand, and R. F. Massung. 2003. Detection of *Borrelia burgdorferi*, *Ehrlichia chaffeensis*, and *Anaplasma phagocytophilum* in ticks (Acari: Ixodidae) from a coastal region of California. Journal of Medical Entomology 40:534–539.

Persing, D. H., D. Mathiesen, W. F. Marshall, S. R. Telford, A. Spielman, J. W. Thomford, and P. A. Conrad. 1992. Detection of *Babesia microti* by polymerase chain reaction. Journal of Clinical Microbiology 30:2097–2103.

Sun, Y., Sriramajayam, K., Luo, D., & Liao, D. (2012). A quick, cost-free method of purification of DNA fragments from agarose gel. *Journal of Cancer*, *3*, 93-95. doi: 10.7150/jca.4163

Tessier, N., Noël, S., & Lapointe, F.-J. (2004). A new method to discriminate the deer mouse (*Peromyscus maniculatus*) from the white-footed mouse (*Peromyscus leucopus*) using species-specific primers in multiplex PCR. *Canadian Journal of Zoology*, *82*(11), 1832–1835. doi: 10.1139/z04-173

Wills, M., Kirby, A., & Lloyd, V. (2018). Detecting the Lyme disease spirochete, *Borrelia burgdorferi*, in ticks using nested PCR. *Journal of Visualized Experiments*, *132*. doi: 10.3791/56471

Wodecka, B. 2011. *flaB* gene as a molecular marker for distinct identification of *Borrelia* species in environmental samples by the PCR-restriction fragment length polymorphism method. Applied and Environmental Microbiology 77:7088–7092.

Zinck, C., J. Priest, D. Shutler, M. Boudreau, and V. Lloyd. 2021. Detection of *Borrelia* spp., *Ehrlichia canis*, *Anaplasma phagocytophilum*, and *Dirofilaria immitis* in eastern coyotes (*Canis latrans*) in Nova Scotia, Canada. Journal of Wildlife Diseases 57:678–682.

**2. Supplementary Tables**

**Supplementary Table 1.** Estimated site-level Lyme disease risk based on the 2018 Lyme disease risk maps in Ontario and Quebec as well as the local abundances and life stages of questing and feeding *Ixodes scapularis*. In Ontario, estimated risk areas are calculated as a 20 km radius from the centre location of questing *I. scapularis* found through tick dragging (Public Health Ontario 2018). In Quebec, municipality risk levels were associated with human Lyme disease cases as well as the abundances and life stages of *I. scapularis* ticks detected through passive and active surveillance (Institut national de santé publique du Québec 2018). Due to significant differences in the provincial classifications of Lyme disease risk areas, we provide two variables related to estimated local Lyme disease risk: (1) a binary variable (0 = possible risk, 1 = present risk) based on definitions by Public Health Ontario and (2) a 3-category variable (1 = possible risk, 2 = present risk, 3 = significant risk) based on definitions by Institut national de santé publique du Québec.

| Site ID | Site | Estimated Lyme disease risk  (possible or present) | Estimated Lyme disease risk  (possible, present, or significant) |
| --- | --- | --- | --- |
| 1 | 3 Ridges Farm | 0 | 1 |
| 2 | New New Age Farm | 1 | 3 |
| 3 | North Tract | 1 | 2 |
| 4 | Brown Hill Tract | 1 | 2 |
| 5 | Upjohn Nature Reserve | 0 | 1 |
| 6 | Dyer Memorial Nature Reserve | 0 | 1 |
| 7 | Rose Hill Nature Reserve | 0 | 1 |
| 8 | Kirkview Farm | 1 | 2 |
| 9 | Saint-Polycarpe | 1 | 2 |
| 10 | Saint-Valentin | 1 | 3 |
| 11 | Henryville | 1 | 3 |
| 12 | Lefebvre | 1 | 2 |
| 13 | Parc du Sanctuaire Saint-Majorique | 1 | 3 |
| 14 | Serpentine-de-Coleraine Ecological Reserve | 1 | 2 |
| 15 | Frontenac National Park | 1 | 2 |
| 16 | Saint-Sylvestre | 1 | 2 |

| Questing *I. scapularis* abundance | Feeding *I. scapularis* abundance | Life stage(s) present |
| --- | --- | --- |
| 0 | 0 | None |
| 131 | 33 | Larvae and nymphs |
| 0 | 2 | Nymphs |
| 0 | 0 | None |
| 0 | 0 | None |
| 0 | 0 | None |
| 0 | 0 | None |
| 4 | 1 | Nymphs |
| 1 | 1 | Larvae and nymphs |
| 99 | 18 | Larvae and nymphs |
| 118 | 7 | Larvae, nymphs, and adults |
| 0 | 0 | None |
| 26 | 3 | Larvae and nymphs |
| 3 | 0 | Larvae and nymphs |
| 0 | 0 | None |
| 0 | 0 | None |

**References**

Institut national de santé publique du Québec. 2018. Carte de risque d’acquisition de la maladie de Lyme selon les municipalités du Québec, 2018. Gouvernement du Québec.

Public Health Ontario. 2018. Ontario Lyme disease map 2018: Estimated risk areas. Queen’s Printer for Ontario.

**Supplementary Table 2.** List of small mammal specimens collected during our field surveys and accessioned at the Redpath Museum, McGill University (Montreal, Quebec, Canada).

| Accession number | Species | Site ID | Location | Latitude (ºN) | Longitude (ºW) |
| --- | --- | --- | --- | --- | --- |
| RMMA20210812 | *Parascalops breweri* | 1 | 3 Ridges Ecological Farm | 42.697 | -81.026 |
| RMMA20210824 | *Peromyscus leucopus* | 1 | 3 Ridges Ecological Farm | 42.697 | -81.026 |
| RMMA20210816 | *Peromyscus leucopus* | 2 | New New Age Farm | 42.73 | -80.835 |
| RMMA20210825 | *Napaeozapus insignis* | 2 | New New Age Farm | 42.73 | -80.835 |
| RMMA20210813 | *Napaeozapus insignis* | 2 | New New Age Farm | 42.731 | -80.837 |
| RMMA20210814 | *Peromyscus leucopus* | 2 | New New Age Farm | 42.731 | -80.836 |
| RMMA20210817 | *Napaeozapus insignis* | 2 | New New Age Farm | 42.731 | -80.837 |
| RMMA20210818 | *Peromyscus leucopus* | 2 | New New Age Farm | 42.731 | -80.836 |
| RMMA20210819 | *Napaeozapus insignis* | 2 | New New Age Farm | 42.731 | -80.837 |
| RMMA20210820 | *Peromyscus leucopus* | 2 | New New Age Farm | 42.731 | -80.836 |
| RMMA20210821 | *Peromyscus leucopus* | 2 | New New Age Farm | 42.731 | -80.836 |
| RMMA20210822 | *Napaeozapus insignis* | 2 | New New Age Farm | 42.731 | -80.836 |
| RMMA20210823 | *Napaeozapus insignis* | 2 | New New Age Farm | 42.731 | -80.837 |
| RMMA20210826 | *Parascalops breweri* | 2 | New New Age Farm | 42.731 | -80.835 |
| RMMA20210827 | *Napaeozapus insignis* | 2 | New New Age Farm | 42.731 | -80.837 |
| RMMA20210828 | *Napaeozapus insignis* | 2 | New New Age Farm | 42.731 | -80.837 |
| RMMA20210829 | *Napaeozapus insignis* | 2 | New New Age Farm | 42.731 | -80.837 |
| RMMA20210830 | *Napaeozapus insignis* | 2 | New New Age Farm | 42.731 | -80.837 |
| RMMA20210831 | *Napaeozapus insignis* | 2 | New New Age Farm | 42.731 | -80.837 |
| RMMA20210815 | *Peromyscus leucopus* | 2 | New New Age Farm | 42.771 | -80.835 |
| RMMA20210838 | *Napaeozapus insignis* | 3 | North Tract | 44.081 | -79.311 |
| RMMA20210840 | *Microtus pennsylvanicus* | 3 | North Tract | 44.081 | -79.31 |
| RMMA20210841 | *Napaeozapus insignis* | 3 | North Tract | 44.081 | -79.311 |
| RMMA20210839 | *Napaeozapus insignis* | 3 | North Tract | 44.09 | -79.311 |
| RMMA20210833 | *Napaeozapus insignis* | 3 | North Tract | 44.081 | -79.31 |
| RMMA20210832 | *Peromyscus leucopus* | 3 | North Tract | 44.082 | -79.313 |
| RMMA20210834 | *Peromyscus leucopus* | 4 | Brown Hill Tract | 44.209 | -79.366 |
| RMMA20210835 | *Peromyscus leucopus* | 4 | Brown Hill Tract | 44.209 | -79.366 |
| RMMA20210842 | *Peromyscus leucopus* | 4 | Brown Hill Tract | 44.209 | -79.366 |
| RMMA20210836 | *Peromyscus leucopus* | 4 | Brown Hill Tract | 44.21 | -79.366 |
| RMMA20210837 | *Peromyscus leucopus* | 4 | Brown Hill Tract | 44.21 | -79.366 |
| RMMA20210809 | *Peromyscus leucopus* | 5 | Upjohn Nature Reserve | 45.076 | -79.36 |
| RMMA20210810 | *Peromyscus leucopus* | 5 | Upjohn Nature Reserve | 45.076 | -79.36 |
| RMMA20210808 | *Peromyscus maniculatus* | 6 | Dyer Memorial Nature Reserve | 45.404 | -79.149 |
| RMMA20210811 | *Napaeozapus insignis* | 6 | Dyer Memorial Nature Reserve | 45.404 | -79.149 |
| RMMA20210803 | *Myodes gapperi* | 7 | Rose Hill Nature Reserve | 45.159 | -77.226 |
| RMMA20210806 | *Myodes gapperi* | 7 | Rose Hill Nature Reserve | 45.159 | -77.227 |
| RMMA20210807 | *Peromyscus maniculatus* | 7 | Rose Hill Nature Reserve | 45.159 | -77.227 |
| RMMA20210801 | *Peromyscus maniculatus* | 7 | Rose Hill Nature Reserve | 45.16 | -77.227 |
| RMMA20210802 | *Napaeozapus insignis* | 7 | Rose Hill Nature Reserve | 45.16 | -77.226 |
| RMMA20210804 | *Peromyscus maniculatus* | 7 | Rose Hill Nature Reserve | 45.16 | -77.227 |
| RMMA20210805 | *Napaeozapus insignis* | 7 | Rose Hill Nature Reserve | 45.16 | -77.226 |
| RMMA20210843 | *Peromyscus leucopus* | 8 | Kirkview Farm | 45.422 | -74.67 |
| RMMA20210844 | *Peromyscus leucopus* | 8 | Kirkview Farm | 45.422 | -74.67 |
| RMMA20210846 | *Peromyscus leucopus* | 8 | Kirkview Farm | 45.422 | -74.67 |
| RMMA20210847 | *Peromyscus leucopus* | 8 | Kirkview Farm | 45.422 | -74.67 |
| RMMA20210848 | *Peromyscus leucopus* | 8 | Kirkview Farm | 45.422 | -74.67 |
| RMMA20210852 | *Blarina brevicauda* | 9 | Saint-Polycarpe | 45.329 | -74.394 |
| RMMA20210855 | *Peromyscus leucopus* | 9 | Saint-Polycarpe | 45.329 | -74.394 |
| RMMA20210856 | *Peromyscus leucopus* | 9 | Saint-Polycarpe | 45.329 | -74.394 |
| RMMA20210845 | *Peromyscus leucopus* | 9 | Saint-Polycarpe | 45.33 | -74.394 |
| RMMA20210849 | *Peromyscus leucopus* | 9 | Saint-Polycarpe | 45.33 | -74.394 |
| RMMA20210850 | *Peromyscus leucopus* | 9 | Saint-Polycarpe | 45.33 | -74.394 |
| RMMA20210851 | *Peromyscus leucopus* | 9 | Saint-Polycarpe | 45.33 | -74.394 |
| RMMA20210853 | *Peromyscus leucopus* | 9 | Saint-Polycarpe | 45.33 | -74.394 |
| RMMA20210854 | *Blarina brevicauda* | 9 | Saint-Polycarpe | 45.33 | -74.394 |
| RMMA20210857 | *Peromyscus leucopus* | 9 | Saint-Polycarpe | 45.33 | -74.394 |
| RMMA20210858 | *Blarina brevicauda* | 9 | Saint-Polycarpe | 45.33 | -74.394 |
| RMMA20210859 | *Blarina brevicauda* | 9 | Saint-Polycarpe | 45.33 | -74.394 |
| RMMA20210860 | *Blarina brevicauda* | 9 | Saint-Polycarpe | 45.33 | -74.394 |
| RMMA20210866 | *Peromyscus leucopus* | 10 | Saint-Valentin | 45.185 | -73.348 |
| RMMA20210867 | *Peromyscus leucopus* | 10 | Saint-Valentin | 45.185 | -73.348 |
| RMMA20210868 | *Peromyscus leucopus* | 10 | Saint-Valentin | 45.185 | -73.347 |
| RMMA20210869 | *Myodes gapperi* | 10 | Saint-Valentin | 45.185 | -73.347 |
| RMMA20210870 | *Napaeozapus insignis* | 10 | Saint-Valentin | 45.185 | -73.347 |
| RMMA20210871 | *Myodes gapperi* | 10 | Saint-Valentin | 45.185 | -73.347 |
| RMMA20210872 | *Myodes gapperi* | 10 | Saint-Valentin | 45.185 | -73.347 |
| RMMA20210874 | *Myodes gapperi* | 10 | Saint-Valentin | 45.185 | -73.348 |
| RMMA20210875 | *Myodes gapperi* | 10 | Saint-Valentin | 45.185 | -73.347 |
| RMMA20210861 | *Peromyscus maniculatus* | 11 | Henryville | 45.117 | -73.212 |
| RMMA20210862 | *Peromyscus maniculatus* | 11 | Henryville | 45.117 | -73.212 |
| RMMA20210864 | *Peromyscus maniculatus* | 11 | Henryville | 45.117 | -73.21 |
| RMMA20210873 | *Myodes gapperi* | 11 | Henryville | 45.117 | -73.211 |
| RMMA20210863 | *Peromyscus leucopus* | 11 | Henryville | 45.118 | -73.211 |
| RMMA20210865 | *Peromyscus leucopus* | 11 | Henryville | 45.118 | -73.211 |
| RMMA20210883 | *Myodes gapperi* | 12 | Lefebvre | 45.738 | -72.406 |
| RMMA20210884 | *Myodes gapperi* | 12 | Lefebvre | 45.738 | -72.406 |
| RMMA20210885 | *Blarina brevicauda* | 12 | Lefebvre | 45.738 | -72.406 |
| RMMA20210886 | *Blarina brevicauda* | 12 | Lefebvre | 45.738 | -72.406 |
| RMMA20210892 | *Blarina brevicauda* | 12 | Lefebvre | 45.738 | -72.406 |
| RMMA20210888 | *Myodes gapperi* | 13 | Parc du Sanctuaire Saint-Majorique | 45.943 | 72.53 |
| RMMA20210891 | *Napaeozapus insignis* | 13 | Parc du Sanctuaire Saint-Majorique | 45.943 | -72.529 |
| RMMA20210893 | *Blarina brevicauda* | 13 | Parc du Sanctuaire Saint-Majorique | 45.943 | -72.53 |
| RMMA20210894 | *Myodes gapperi* | 13 | Parc du Sanctuaire Saint-Majorique | 45.943 | -72.531 |
| RMMA20210881 | *Peromyscus maniculatus* | 13 | Parc du Sanctuaire Saint-Majorique | 45.944 | -72.53 |
| RMMA20210882 | *Myodes gapperi* | 13 | Parc du Sanctuaire Saint-Majorique | 45.944 | -72.531 |
| RMMA20210887 | *Napaeozapus insignis* | 13 | Parc du Sanctuaire Saint-Majorique | 45.944 | -72.53 |
| RMMA20210889 | *Napaeozapus insignis* | 13 | Parc du Sanctuaire Saint-Majorique | 45.944 | -72.53 |
| RMMA20210890 | *Myodes gapperi* | 13 | Parc du Sanctuaire Saint-Majorique | 45.944 | -72.529 |
| RMMA20210895 | *Napaeozapus insignis* | 13 | Parc du Sanctuaire Saint-Majorique | 45.944 | -72.53 |
| RMMA20210877 | *Peromyscus maniculatus* | 14 | Serpentine-de-Coleraine Ecological Reserve | 45.978 | -71.37 |
| RMMA20210878 | *Peromyscus maniculatus* | 14 | Serpentine-de-Coleraine Ecological Reserve | 45.978 | -71.371 |
| RMMA20210879 | *Myodes gapperi* | 14 | Serpentine-de-Coleraine Ecological Reserve | 45.978 | -71.37 |
| RMMA20210880 | *Peromyscus maniculatus* | 14 | Serpentine-de-Coleraine Ecological Reserve | 45.978 | -71.37 |
| RMMA20210876 | *Sorex cinereus* | 15 | Frontenac National Park | 45.815 | -71.203 |
| RMMA20210896 | *Blarina brevicauda* | 16 | Saint-Sylvestre | 46.368 | -71.119 |
| RMMA20210897 | *Blarina brevicauda* | 16 | Saint-Sylvestre | 46.368 | -71.118 |
| RMMA20210898 | *Blarina brevicauda* | 16 | Saint-Sylvestre | 46.368 | -71.118 |
| RMMA20210899 | *Napaeozapus insignis* | 16 | Saint-Sylvestre | 46.368 | -71.118 |
| RMMA202108100 | *Blarina brevicauda* | 16 | Saint-Sylvestre | 46.368 | -71.118 |
| RMMA202108101 | *Blarina brevicauda* | 16 | Saint-Sylvestre | 46.368 | -71.119 |
| RMMA202108102 | *Myodes gapperi* | 16 | Saint-Sylvestre | 46.368 | -71.119 |
| RMMA202108103 | *Blarina brevicauda* | 16 | Saint-Sylvestre | 46.368 | -71.118 |
| RMMA202108104 | *Blarina brevicauda* | 16 | Saint-Sylvestre | 46.368 | -71.118 |
| RMMA202108105 | *Blarina brevicauda* | 16 | Saint-Sylvestre | 46.368 | -71.118 |

**Supplementary Table 3.** Primers and conditions used in genetic testing of tick and mammal samples with nested PCRs for *Anaplasma phagocytophilum*, *Babesia odocoilei*, *Babesia microti*, *Borrelia burgdorferi*, and *Borrelia miyamotoi*. For pathogen testing workflow, see Supplementary Figures in Crandall et al. 2022.

| Species name | Primer name | Reference | Target gene |
| --- | --- | --- | --- |
| *Anaplasma*  *phagocytophilum* | AnaP44OutL1-F | In house | p44 |
|  | AnaP44OutL1-R |  |  |
|  | AnaP44InF | Holden et al. 2003 |  |
|  | AnaP44InR |  |  |
| *Babesia spp.* | BabGenPCRF | Scott et al. 2021 | 18S rRNA |
|  | BabGenPCRR |  |  |
| *Babesia microti* | Mic494 | In house |  |
|  | BabGenInR1 |  |  |
| *Babesia odocoilei* | Odo563 | In house |  |
|  | BabGenInR1 |  |  |
| *Babesia microti* | BabMicOutF | Persing et al. 1992 | 18S rRNA |
|  | BabMicOutR |  |  |
|  | BabMicInF |  |  |
|  | BabMicInR |  |  |
| *Babesia odocoilei* | Bab1F Out | National Microbiology Lab (Canada) | 18S rRNA |
|  | Bab4R Out |  |  |
|  | Bab2F In |  |  |
|  | Bab3R In |  |  |
| *Borrelia burgdorferi s.l.* | MLP-0035 | Wodecka 2011 | Flagellin B (*flaB*) |
|  | MLP-0036 |  |  |
|  | MLP-0037 |  |  |
|  | MLP-0038 |  |  |
| *Borrelia spp.* | VETTBOROUTF | Dibernardo et al. 2014 | 5S-23S Intergenic Space Region |
|  | VETTBOROUTR |  |  |
|  |  |  |  |
|  |  |  |  |
| *Borrelia burgdorferi s.s.* | VETTBURGINF | Dibernardo et al. 2014 | 5S-23S Intergenic Space Region |
|  | VETTBURGINR |  |  |
| *Borrelia miyamotoi* | VETTMIYINF | Zinck et al. 2021 | 18S rRNA |
|  | VETTMIYINR |  |  |

| Sequence (5’ > 3’) | Amplicon size | Initial Denaturation | | Denaturation | | Annealing | | Elongation | |
| --- | --- | --- | --- | --- | --- | --- | --- | --- | --- |
|  |  | (s) | (°C) | (s) | (°C) | (s) | (°C) | (s) | (°C) |
| gtagaagaaaccgccctaat | 850 | 300 | 95 | 30 | 95 | 30 | 53 | 60 | 72 |
| tctatgttggtttggattacag |  |  |  |  |  |  |  |  |  |
| gcccagtaacaacatcataagc | 334 | 300 | 95 | 30 | 95 | 30 | 53 | 60 | 72 |
| ccagcgtttagcaagataagag |  |  |  |  |  |  |  |  |  |
| gtcttgtaattggaatgatgg | 488 | 300 | 95 | 30 | 95 | 30 | 55 | 45 | 72 |
| tagtttatggttaggactacg |  |  |  |  |  |  |  |  |  |
| ccgtctcggctctttgcc | 308 | 300 | 95 | 30 | 95 | 15 | 63 | 20 | 72 |
| tctgatcgtcttcgatcccc |  |  |  |  |  |  |  |  |  |
| ccgtattttgacttttgtcgactgt | 311 | 300 | 95 | 30 | 95 | 15 | 63 | 20 | 72 |
| tctgatcgtcttcgatcccc |  |  |  |  |  |  |  |  |  |
| cttagtataagcttttatacagc | 238 | 300 | 94 | 30 | 94 | 30 | 55 | 30 | 72 |
| ataggtcagaaacttgaatgataca |  |  |  |  |  |  |  |  |  |
| gttatagtttatttgatgttc | 155 | 300 | 94 | 30 | 94 | 30 | 55 | 30 | 72 |
| aagccatgcgattcgctaat |  |  |  |  |  |  |  |  |  |
| ccgtcgtagtcctaacyataaac | 767 | 300 | 95 | 30 | 95 | 60 | 52 | 60 | 72 |
| ccttgttacgacttctccttcc |  |  |  |  |  |  |  |  |  |
| ttcttgattctytgggtrgtgg | 343 | 300 | 95 | 30 | 95 | 60 | 55 | 60 | 72 |
| ctaggcattcctcgttcawgat |  |  |  |  |  |  |  |  |  |
| tggtatgggagtttctgg | 774 | 600 | 94 | 30 | 94 | 45 | 50 | 60 | 72 |
| tctgtcattgtagcatcttt |  |  |  |  |  |  |  |  |  |
| cagacaacagagggaaat | 605 | 600 | 94 | 30 | 94 | 45 | 54 | 60 | 72 |
| tcaagtctattttggaaagcacc |  |  |  |  |  |  |  |  |  |
| gtatgtttagtgaggggggtg | 1029 (*B. burgdorferi*);  588 (*B. miyamotoi*) | 240 | 94 | 60 | 94 | 60 | 50 | 60 | 72 |
| ggatcatagctcaggtggttag |  |  |  |  |  |  |  |  |  |
|  |  |  |  |  |  |  |  |  |  |
|  |  |  |  |  |  |  |  |  |  |
|  |  |  |  |  |  |  |  |  |  |
|  |  |  |  |  |  |  |  |  |  |
|  |  |  |  |  |  |  |  |  |  |
| atgtattccattgttttaattacg | 340 | 300 | 95 | 30 | 95 | 30 | 51 | 30 | 72 |
| gacaagtattgtagcgagc |  |  |  |  |  |  |  |  |  |
| ataaacctgaggtcggagg | 507 | 300 | 95 | 30 | 95 | 30 | 60 | 30 | 72 |
| aaagtgtggctggatcacc |  |  |  |  |  |  |  |  |  |

| Repetitions | Final Elongation | |
| --- | --- | --- |
|  | (s) | (°C) |
| 35 | 300 | 72 |
| 40 | 600 | 72 |
| 40 | 600 | 72 |
| 40 | 600 | 72 |
| 40 | 600 | 72 |
| 30 | 300 | 72 |
| 30 | 300 | 72 |
| 30 | 600 | 72 |
| 30 | 600 | 72 |
| 40 | 420 | 72 |
| 40 | 420 | 72 |
| 35 | 600 | 72 |
|  |  |  |
| 40 | 600 | 72 |
| 40 | 600 | 72 |

**References**

Crandall, K. E., J. T. Kerr, and V. Millien. 2022. Emerging tick-borne pathogens in Central Canada: Recent detections of *Babesia odocoilei* and *Rickettsia rickettsii*. Vector-Borne and Zoonotic Diseases 22:535–544.

Dibernardo, A., T. Cote, N. Ogden, and L. Lindsay. 2014. The prevalence of *Borrelia miyamotoi* infection, and co-infections with other *Borrelia* spp. in *Ixodes scapularis* ticks collected in Canada. Parasites & Vectors 7.

Holden, K., J. T. Boothby, S. Anand, and R. F. Massung. 2003. Detection of *Borrelia burgdorferi*, *Ehrlichia chaffeensis*, and *Anaplasma phagocytophilum* in ticks (Acari: Ixodidae) from a coastal region of California. Journal of Medical Entomology 40:534–539.

Persing, D. H., D. Mathiesen, W. F. Marshall, S. R. Telford, A. Spielman, J. W. Thomford, and P. A. Conrad. 1992. Detection of *Babesia microti* by polymerase chain reaction. Journal of Clinical Microbiology 30:2097–2103.

Scott, J., E. Pascoe, M. Sajid, and J. Foley. 2021. Detection of *Babesia odocoilei* in *Ixodes scapularis* ticks collected in Southern Ontario, Canada. Pathogens 10:327.

Wodecka, B. 2011. *flaB* gene as a molecular marker for distinct identification of *Borrelia* species in environmental samples by the PCR-restriction fragment length polymorphism method. Applied and Environmental Microbiology 77:7088–7092.

Zinck, C., J. Priest, D. Shutler, M. Boudreau, and V. Lloyd. 2021. Detection of *Borrelia* spp., *Ehrlichia canis*, *Anaplasma phagocytophilum*, and *Dirofilaria immitis* in eastern coyotes (*Canis latrans*) in Nova Scotia, Canada. Journal of Wildlife Diseases 57:678–682.

**Supplementary Table 4.** Summary of collected small mammals at our sites in Ontario and Quebec, Canada, where the abundance of infected and total small mammal individuals are indicated. Only two small mammals were infected with pathogens across our sites including one *Peromyscus leucopus* (*Babesia microti*) and one *Blarina brevicauda* (*Babesia odocoilei*).

| Site ID | Site | Collected specimens | | | | | | |
| --- | --- | --- | --- | --- | --- | --- | --- | --- |
|  |  | *P. leucopus* | *P. maniculatus* | *B. brevicauda* | *S. cinereus* | *M. pennsylvanicus* | *M. gapperi* |  |
| 1 | 3 Ridges Farm | 0/1 | 0 | 0 | 0 | 0 | 0 |  |
| 2 | New New Age Farm | 0/6 | 0 | 0 | 0 | 0 | 0 |  |
| 3 | North Tract | 0/1 | 0 | 0 | 0 | 0/1 | 0 |  |
| 4 | Brown Hill Tract | 0/5 | 0 | 0 | 0 | 0 | 0 |  |
| 5 | Upjohn Nature Reserve | 0/2 | 0 | 0 | 0 | 0 | 0 |  |
| 6 | Dyer Memorial Nature Reserve | 0 | 0/1 | 0 | 0 | 0 | 0 |  |
| 7 | Rose Hill Nature Reserve | 0 | 0/3 | 0 | 0 | 0 | 0/2 |  |
| 8 | Kirkview Farm | 0/5 | 0 | 0 | 0 | 0 | 0 |  |
| 9 | Saint-Polycarpe | 0/8 | 0 | 0/5 | 0 | 0 | 0 |  |
| 10 | Saint-Valentin | 0/3 | 0 | 0 | 0 | 0 | 0/5 |  |
| 11 | Henryville | 1/2 | 0/3 | 0 | 0 | 0 | 0/1 |  |
| 12 | Lefebvre | 0 | 0 | 0/3 | 0 | 0 | 0/2 |  |
| 13 | Parc du Sanctuaire Saint-Majorique | 0 | 0/1 | 0/1 | 0 | 0 | 0/4 |  |
| 14 | Serpentine-de-Coleraine Ecological Reserve | 0 | 0/3 | 0 | 0 | 0 | 0/1 |  |
| 15 | Frontenac National Park | 0 | 0 | 0 | 0/1 | 0 | 0 |  |
| 16 | Saint-Sylvestre | 0 | 0 | 1/8 | 0 | 0 | 0/1 |  |

|  | | Pathogen(s) detected |
| --- | --- | --- |
| *P. breweri* | *N. insignis* |  |
| 0/1 | 0 | None |
| 0/1 | 0/11 | None |
| 0 | 0/4 | None |
| 0 | 0 | None |
| 0 | 0 | None |
| 0 | 0/1 | None |
| 0 | 0/2 | None |
| 0 | 0 | None |
| 0 | 0 | None |
| 0 | 0/1 | None |
| 0 | 0 | *B. microti* (*P. leucopus*) |
| 0 | 0 | None |
| 0 | 0/4 | None |
| 0 | 0 | None |
| 0 | 0 | None |
| 0 | 0/1 | *B. odocoilei* (*B. brevicauda*) |

**Supplementary Table 5.** Summary of the released small mammal species at our sites in Ontario and Quebec, Canada. The presence of released small mammals including juveniles and non-targeted species is indicated with an “X”.

| Site ID | Site | *Peromyscus spp.* | *B. brevicauda* | *N. insignis* | *G. volans* | *S. carolinensis* | *T. striatus* | *T. hudsonicus* |
| --- | --- | --- | --- | --- | --- | --- | --- | --- |
| 1 | 3 Ridges Farm | - | - | - | X | - | X | - |
| 2 | New New Age Farm | X | - | - | - | - | X | - |
| 3 | North Tract | X | - | X | - | - | X | - |
| 4 | Brown Hill Tract | X | - | - | - | - | - | - |
| 5 | Upjohn Nature Reserve | X | - | - | X | - | - | - |
| 6 | Dyer Memorial Nature Reserve | - | - | - | - | - | - | - |
| 7 | Rose Hill Nature Reserve | - | - | - | X | - | - | - |
| 8 | Kirkview Farm | - | - | - | - | - | X | - |
| 9 | Saint-Polycarpe | X | X | - | - | - | X | - |
| 10 | Saint-Valentin | X | X | - | - | - | - | - |
| 11 | Henryville | X | X | - | - | - | X | - |
| 12 | Lefebvre | X | - | - | - | - | - | - |
| 13 | Parc du Sanctuaire Saint-Majorique | X | - | - | X | X | - | X |
| 14 | Serpentine-de-Coleraine Ecological Reserve | X | - | - | - | - | - | - |
| 15 | Frontenac National Park | X | - | - | - | - | X | - |
| 16 | Saint-Sylvestre | X | X | X | - | - | - | X |

**Supplementary Table 6.** The abundance of infected and total questing and feeding *Ixodes scapularis* at our sites in Ontario and Quebec, Canada, with infection prevalence in parentheses. Tick abundance included pools of larvae separated by grids (questing) and hosts (feeding), individual nymphs, and individual adults. No questing or feeding ticks tested positive for *Anaplasma phagocytophilum*, *Babesia microti*, or *Borrelia miyamotoi*.

| Site ID | Site | *Babesia odocoilei* | | *Borrelia burgdorferi* | |
| --- | --- | --- | --- | --- | --- |
|  |  | Questing | Feeding | Questing | Feeding |
| 1 | 3 Ridges Farm | 0 | 0 | 0 | 0 |
| 2 | New New Age Farm | 1/27 (3.7%) | 0/9 (0%) | 1/27 (3.7%) | 0/9 (0%) |
| 3 | North Tract | 0 | 0/2 (0%) | 0 | 0/2 (0%) |
| 4 | Brown Hill Tract | 0 | 0 | 0 | 0 |
| 5 | Upjohn Nature Reserve | 0 | 0 | 0 | 0 |
| 6 | Dyer Memorial Nature Reserve | 0 | 0 | 0 | 0 |
| 7 | Rose Hill Nature Reserve | 0 | 0/1 (0%) | 0 | 0/1 (0%) |
| 8 | Kirkview Farm | 0/4 (0%) | 0/1 (0%) | 0/4 (0%) | 1/1 (100%) |
| 9 | Saint-Polycarpe | 0/1 (0%) | 0/1 (0%) | 0/1 (0%) | 0/1 (0%) |
| 10 | Saint-Valentin | 1/71 (1.4%) | 0/5 (0%) | 18/71 (25.4%) | 0/5 (0%) |
| 11 | Henryville | 1/37 (2.7%) | 1/5 (20.0%) | 3/37 (8.1%) | 0/5 (0%) |
| 12 | Lefebvre | 0/1 (0%) | 0 | 0/1 (0%) | 0 |
| 13 | Parc du Sanctuaire Saint-Majorique | 2/14 (14.3%) | 0/3 (0%) | 0/14 (0%) | 1/3 (33.3%) |
| 14 | Serpentine-de-Coleraine Ecological Reserve | 0/22 (0%) | 0 | 0/22 (0%) | 0 |
| 15 | Frontenac National Park | 0/1 (0%) | 0 | 0/1 (0%) | 0 |
| 16 | Saint-Sylvestre | 0/1 (0%) | 0 | 0/1 (0%) | 0 |

**Supplementary Table 7.** The abundance of infected and total small mammal hosts at our sites in Ontario and Quebec, Canada, with infection prevalence in parentheses. No small mammal hosts tested positive for *Anaplasma phagocytophilum*, *Borrelia burgdorferi*, or *Borrelia miyamotoi*.

| Site ID | Site | *Babesia odocoilei* | *Babesia microti* |
| --- | --- | --- | --- |
| 1 | 3 Ridges Farm | 0/2 (0%) | 0/2 (0%) |
| 2 | New New Age Farm | 0/18 (0%) | 0/18 (0%) |
| 3 | North Tract | 0/6 (0%) | 0/6 (0%) |
| 4 | Brown Hill Tract | 0/5 (0%) | 0/5 (0%) |
| 5 | Upjohn Nature Reserve | 0/2 (0%) | 0/2 (0%) |
| 6 | Dyer Memorial Nature Reserve | 0/2 (0%) | 0/2 (0%) |
| 7 | Rose Hill Nature Reserve | 0/7 (0%) | 0/7 (0%) |
| 8 | Kirkview Farm | 0/5 (0%) | 0/5 (0%) |
| 9 | Saint-Polycarpe | 0/13 (0%) | 0/13 (0%) |
| 10 | Saint-Valentin | 0/9 (0%) | 0/9 (0%) |
| 11 | Henryville | 0/6 (0%) | 1/6 (16.7%) |
| 12 | Lefebvre | 0/5 (0%) | 0/5 (0%) |
| 13 | Parc du Sanctuaire Saint-Majorique | 0/10 (0%) | 0/10 (0%) |
| 14 | Serpentine-de-Coleraine Ecological Reserve | 0/4 (0%) | 0/4 (0%) |
| 15 | Frontenac National Park | 0/1 (0%) | 0/1 (0%) |
| 16 | Saint-Sylvestre | 1/10 (10%) | 0/10 (0%) |

**Supplementary Table 8.** Parameter estimates for the binomial generalized linear mixed models assessing the impact of the abundance of questing and feeding *Ixodes scapularis* ticks on pathogen presence across our sites in Ontario and Quebec, Canada. Pathogen presence indicated whether pathogens were present (1) or absent (0) in *I. scapularis* or in small mammal specimens at a locality. All biotic factors were centred and standardized. A random factor of Site was also included in the models. The model formula for *I. scapularis* ticks is Pathogen presence (0/1) ~ abundance of *I. scapularis* ticks + (1|Site). Significance of terms is denoted by *** *P* < 0.001, ** *P* < 0.01, * *P* < 0.05.

*Binomial – cloglog link function* (AIC: 14.71)

|  | Estimate | Standard error | *z* value | *P* value |
| --- | --- | --- | --- | --- |
| Intercept | 14.050 | 10.500 | 1.339 | 0.181 |
| *I. scapularis* abundance | 32.560 | 22.260 | 1.462 | 0.144 |

Site – Variance = 0, Std. Dev = 0

*Binomial – logit link function* (AIC: 14.98)

|  | Estimate | Standard error | *z* value | *P* value |
| --- | --- | --- | --- | --- |
| Intercept | 14.360 | 12.260 | 1.171 | 0.242 |
| *I. scapularis* abundance | 32.910 | 25.580 | 1.287 | 0.198 |

Site – Variance < 0.001, Std. Dev < 0.001

**Supplementary Table 9.** Parameter estimates for the binomial generalized linear mixed models assessing the impact of the relative abundance of *Peromyscus leucopus* and mammal species richness on pathogen presence across our sites in Ontario and Quebec, Canada. Pathogen presence indicated whether pathogens were present (1) or absent (0) in *I. scapularis* ticks or in small mammal specimens at a locality. All biotic factors were centred and standardized. A random factor of Site was also included in the models. The model formula for mammal hosts is Pathogen presence (0/1) ~ Relative abundance *P. leucopus* + No. mammal species + (1|Site). Significance of terms is denoted by *** *P* < 0.001, ** *P* < 0.01, * *P* < 0.05.

*Binomial – cloglog link function* (AIC: 20.49)

|  | Estimate | Standard error | *z* value | *P* value |
| --- | --- | --- | --- | --- |
| Intercept | -1.353 | 0.839 | -1.614 | 0.107 |
| Relative abundance *P. leucopus* | 0.114 | 0.718 | 0.159 | 0.874 |
| No. mammal species | 1.939 | 1.485 | 1.306 | 0.192 |

Site – Variance = 0.405, Std. Dev = 0.636

*Binomial – logit link function* (AIC: 15.19)

|  | Estimate | Standard error | *z* value | *P* value |
| --- | --- | --- | --- | --- |
| Intercept | -11.436 | 5.098 | -2.243 | 0.024* |
| Relative abundance *P. leucopus* | 0.342 | 4.162 | 0.082 | 0.934 |
| No. mammal species | 44.440 | 14.658 | 3.032 | 0.002** |

Site – Variance = 10795, Std. Dev = 103.9

**Supplementary Table 10.** Parameter estimates for the binomial generalized linear mixed model assessing the effect of the relative abundance of *Peromyscus leucopus* and mammal species richness on pathogen prevalence in questing *Ixodes scapularis* ticks across our sites in Ontario and Quebec, Canada. All biotic factors were centred and standardized. In addition, a random factor of Site was included in the model. The model formula is Pathogen prevalence ~ Relative abundance *P. leucopus* + No. mammal species + (1|Site). Significance of terms is denoted by *** *P* < 0.001, ** *P* < 0.01, * *P* < 0.05.

*Binomial – cloglog link function* (AIC: 28.15)

|  | Estimate | Standard error | *z* value | *P* value |
| --- | --- | --- | --- | --- |
| Intercept | -1.813 | 0.421 | -4.305 | < 0.001 |
| Relative abundance *P. leucopus* | -0.111 | 0.498 | -0.224 | 0.824 |
| No. mammal species | 0.294 | 0.990 | 0.297 | 0.767 |

Site – Variance = 0.1289, Std. Dev = 0.359

*Binomial – logit link function* (AIC: 28.15)

|  | Estimate | Standard error | *z* value | *P* value |
| --- | --- | --- | --- | --- |
| Intercept | -1.718 | 0.461 | -3.724 | < 0.001 |
| Relative abundance *P. leucopus* | -0.112 | 0.550 | -0.204 | 0.838 |
| No. mammal species | 0.337 | 1.109 | 0.304 | 0.761 |

Site – Variance = 0.1507, Std. Dev = 0.3882

**Supplementary Table 11.** Parameter estimates for the ordinal logistic regression assessing the effect of the relative abundance of *Peromyscus leucopus* and mammal species richness on the number of pathogen species detected in *Ixodes scapularis* and small mammal specimens across our sites in Ontario and Quebec, Canada. The number of pathogen species (or pathogen diversity) ranged from 0 to 3 distinct species, which represents the four levels of our ordinal variable. All biotic factors were centred and standardized. The model formula is Pathogen diversity ~ Relative abundance *P. leucopus* + No. mammal species. One additional model was assessed after employing the *stepAIC* function from the *cAIC4* package on our full model (Säfken et al. 2021). The additional model was Pathogen diversity ~ No. mammal species. Significance of terms is denoted by *** *P* < 0.001, ** *P* < 0.01, * *P* < 0.05.

|  | Estimate | Standard error | *t* value | *P* value |
| --- | --- | --- | --- | --- |
| Relative abundance *P. leucopus* | 0.143 | 0.633 | 0.226 | 0.822 |
| No. mammal species | 2.485 | 1,055 | 2.355 | 0.019* |
| 0\|1 | 1.088 | 0.818 | 1.330 | 0.184 |
| 1\|2 | 2.160 | 0.980 | 2.205 | 0.028 |
| 2\|3 | 4.540 | 1.593 | 2.850 | 0.004** |

AIC: 33.152, residual deviance = 23.152

|  | Estimate | Standard error | *t* value | *P* value |
| --- | --- | --- | --- | --- |
| No. mammal species | 2.470 | 1.056 | 2.339 | 0.019* |
| 0\|1 | 1.079 | 0.810 | 1.333 | 0.183 |
| 1\|2 | 2.147 | 0.973 | 2.206 | 0.027 |
| 2\|3 | 4.559 | 1.606 | 2.839 | 0.004** |

AIC: 31.203, residual deviance = 23.202

**References**

Säfken B, Rügamer D, Kneib T, Greven S (2021). “Conditional Model Selection in Mixed-Effects Models with cAIC4.” Journal of Statistical Software, 99(8), 1-30. doi:10.18637/jss.v099.i08

**3. Supplementary Figure**

**
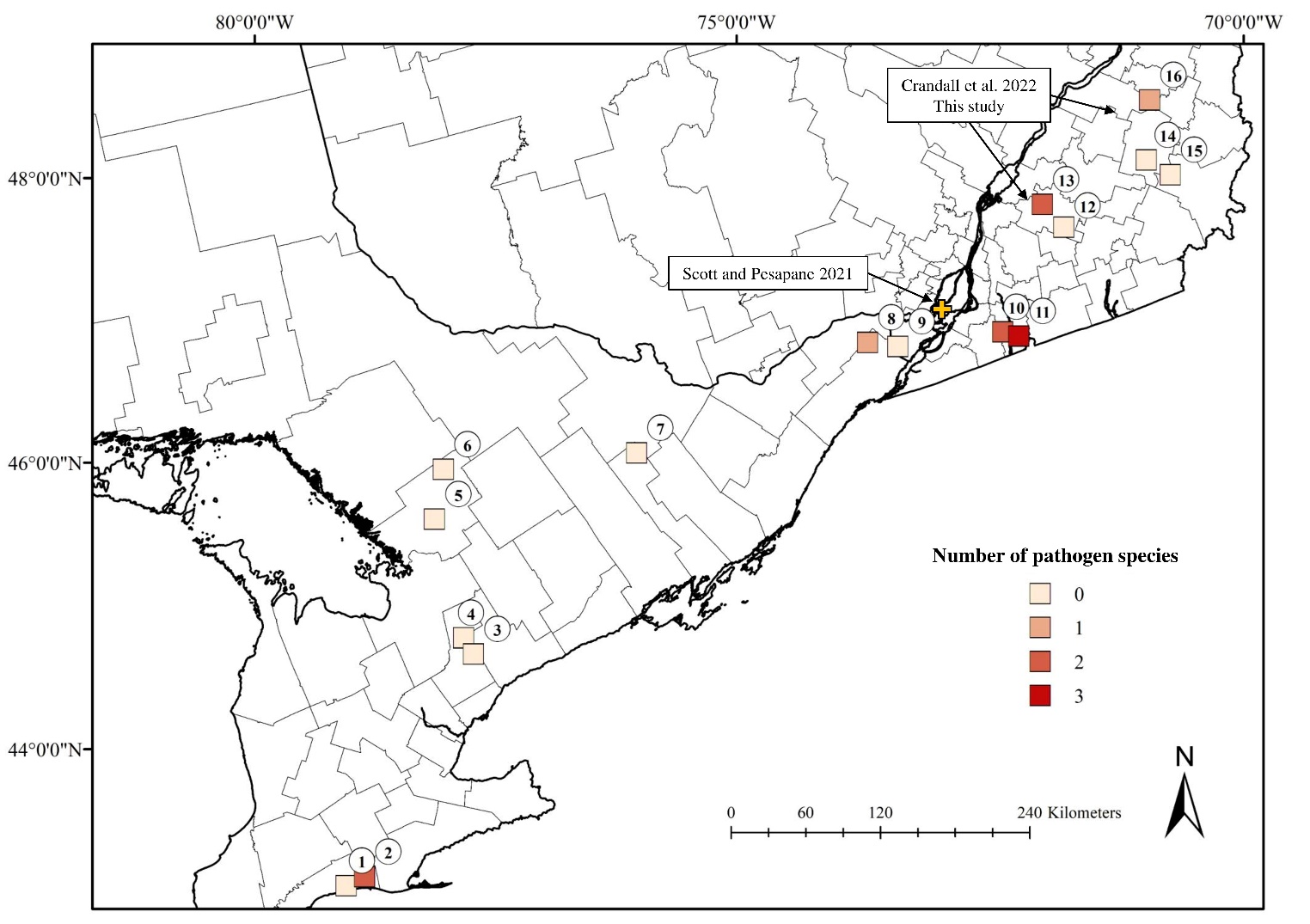
**

**Supplementary Figure 1.** The range limits of *Babesia odocoilei* in Quebec, Canada. Scott and Pesapane (2021) found that *B. odocoilei* was present in *Ixodes scapularis* at Sainte-Anne-de-Bellevue, which previously represented the most poleward detection of this

pathogen in Quebec. This study demonstrates that *B. odocoilei* is located furthernorth than previously detected, with two questing

*I. scapularis* nymphs and one shrew (*Blarina brevicauda*) testing positive for the pathogen near Saint-Majorique-de-Grantham (Site 13) and Saint-Sylvestre (Site 16), respectively.

**References**

Crandall, K. E., J. T. Kerr, and V. Millien. 2022. Emerging tick-borne pathogens in Central Canada: Recent detections of *Babesia odocoilei* and *Rickettsia rickettsii*. Vector-Borne and Zoonotic Diseases 22:535–544.

Scott, J. D., and R. R. Pesapane. 2021. Detection of *Anaplasma phagocytophilum*, *Babesia odocoilei*, *Babesia* sp., *Borrelia burgdorferi* sensu lato, and *Hepatozoon canis* in *Ixodes scapularis* ticks collected in Eastern Canada. Pathogens 10:1265.
